# Experimental Study on High-Speed Milling of SiCf/SiC Composites with PCD and CVD Diamond Tools

**DOI:** 10.3390/ma14133470

**Published:** 2021-06-22

**Authors:** Bin Zhang, Yanan Du, Hanliang Liu, Lianjia Xin, Yinfei Yang, Liang Li

**Affiliations:** 1 Beijing Spacecrafts, Beijing 100094, China; zhangbin992626@163.com (B.Z.); lewish@126.com (H.L.); 2 College of Mechanical and Electrical Engineering, Nanjing University of Aeronautics and Astronautics, Nanjing 210016, China; duyanan@nuaa.edu.cn (Y.D.); xinlj@nuaa.edu.cn (L.X.); liliang@nuaa.edu.cn (L.L.)

**Keywords:** SiC_f_/SiC composite, cryogenic machining, PCD tool, CVD diamond tool, tool wear

## Abstract

Silicon carbide fiber reinforced silicon carbide ceramic matrix composite (SiC_f_/SiC composite) is characterized by a high strength-to-density ratio, high hardness, and high temperature resistance. However, due to the brittleness of the matrix material and the anisotropy of the reinforcing phase, it is a huge challenge for machining of the material. The milling method has advantages of a high material removal rate and applicability to complex surface geometry. However, no published literature on milling of SiC_f_/SiC composite has been found up to now. In this paper, high-speed milling of SiC_f_/SiC composites was carried out under dry conditions and cryogenic cooling using liquid nitrogen, respectively. Polycrystalline diamond (PCD) and chemical vapor deposition (CVD) diamond cutting tools were used for the milling work. The cutting performance of the two kinds of tools in high-speed milling of SiC_f_/SiC composites was studied. Tool failure modes and mechanisms were analyzed. The effects of the cooling approach on tool wear and machined surface quality were also investigated. The experimental results showed that under identical cutting parameters and cooling approaches, the PCD tool yielded better cutting performance in terms of a longer tool life and better surface quality than that of the CVD diamond tool. In dry machining, the failure modes of the CVD diamond tool were a large area of spalling on the rake face, edge chipping and severe tool nose fracture, whereas for the PCD tool, only a small area of spalling around the tool nose took place. Compared to the dry machining, the wear magnitudes of both PCD and CVD diamond tools were decreased in cryogenic machining. Additionally, the surface quality also showed significant improvements. This study indicates that the PCD tool is highly suitable for machining of SiC_f_/SiC composite, and that the cryogenic method can improve machining efficiency and surface quality.

## 1. Introduction

With continuous development in science and technology, the requirements of product-making in the fields of automobile, aerospace, and others are becoming tougher and tougher. The traditional materials can no longer meet the requirements of the modern-day applications. Due to their excellent mechanical properties, such as high hardness, high strength (tensile strength higher than 200 MPa, flexural strength higher than 400 MPa), high specific strength (1/3 to 1/4 specific gravity of titanium alloy), high elastic modulus (up to 200 GPa) and high temperature resistance (higher than 1500 °C), fiber reinforced ceramic matrix composites are widely used in industry, especially in the field of aerospace [1,2,3,4,5]. Among them, silicon carbide fiber-reinforced silicon carbide ceramic matrix composite (SiC_f_/SiC composite) is a kind of composite material which is formed by implanting silicon carbide fibers into a silicon carbide matrix.

Even though parts and components made of SiC_f_/SiC composites could be manufactured with a near net shape forming process, subtractive processes such as cutting and grinding are still needed to remove additional materials. However, due to the complex woven structure of the composite material as well as its excellent mechanical properties, it has brought challenges to the machining of SiC_f_/SiC composites. The mechanical machining of SiC_f_/SiC composites is characterized by severe tool wear and short tool life due to the abrasive wear from hard particles, scratch from SiC fiber, and high process temperature [6,7,8]. In addition, deteriorated surface with defects in terms of cavities, micro-cracks, edge chipping and delamination were also found, which was attributed to the anisotropy and heterogeneity of the material.

At present, the machining methods of fiber-reinforced SiC ceramic matrix composites mainly consist of non-traditional machining methods (such as laser machining, abrasive waterjet machining, electrical discharge machining, etc.), conventional machining methods (such as milling, drilling, grinding, etc.), and hybrid machining methods (such as ultrasonic vibration-assisted machining, laser-assisted machining, etc.). Pulsed laser machining of SiC_f_/SiC composites has attracted wide attention due to the unique properties of laser in precision machining [9,10,11,12]. Liu et al. [9] studied femtosecond laser processing of SiC_f_/SiC composites. The effects of process parameters such as the laser power, overlap ratio, and processing steps on the geometry and surface quality of the machined holes were investigated. It was shown that better surface quality was achieved with higher laser power. However, debris was formed and distributed around the hole, and the roundness of the hole was relatively low. Liu et al. [13] carried out experimental investigation and optimization of micro-hole machining with a fiber laser trepan drilling process on C_f_/SiC composite. A comprehensive analysis method was presented on fiber laser trepan drilling to minimize the taper and heat-affected zone (HAZ). Defocusing the amount of laser process had significant influence on the taper. Chen et al. [14] studied the laser-induced ablation mechanisms of SiC_f_/SiC composites irradiated by a continuous-wave laser and pulsed laser. The relationship between laser parameters and ablation depth was analyzed. Under laser irradiation, recrystallized SiC and amorphous SiO_2_ were produced, which were powdery and porous. The depth of laser-induced ablation could be adjusted and controlled by adjusting the laser parameters. Chou et al. [15] carried out an experimental study on micro abrasive jet machining of SiC_f_/SiC composites. It was indicated that the abrasive particle size had a significant effect on the material removal rate. Cracks were found at the interface between fibers. Wei et al. [16] studied electrical discharge machining (EDM) of SiC_f_/SiC composites using electrode vibration and dielectric deep flushing technologies. It was demonstrated that low duty ratio and high gap voltage could improve the material removal rate and debris evacuation efficiency. Yue et al. [17] investigated the effects of thermal stress on material removal of C_f_/SiC composite in EDM. It demonstrated that the removal mechanisms of carbon fiber were both fracture removal on the vertical surface, and spalling removal on the cross-section. Brittle fracture of the SiC matrix was observed. Additionally, an extremely high thermal stress was generated on the discharge surface of the composite, which exceeded the tensile strength of carbon fiber and SiC matrix.

Conventional machining is still a key process to obtain a high material removal rate and fine surface finish of fiber reinforced SiC ceramic matrix composites. A grinding process with diamond abrasive wheels is the mostly used approach to obtain high surface quality [18,19]. Yin et al. [18] investigated the influences of grinding parameters on material removal of SiC_f_/SiC composites. The results showed that the embrittlement of material and enhancement of fiber breakage took place when employing high grinding speed. In addition, the surface quality and efficiency were increased by improving the grinding speed. Zhang et al. [20] revealed the factors influencing the grinding force in surface grinding of unidirectional C_f_/SiC composites. A grinding force model was established based on the multiple-exponential function method. The results showed that due to the anisotropy of fibers, the grinding force had obvious regularity. Moreover, the feed speed had the most significant influence on grinding force. The material removal mechanisms included the fiber breakage, matrix cracking and interface debonding. The minimum quantity lubrication (MQL) technique was applied to the grinding of fiber reinforced ceramic matrix composites to improve the materials’ machinability. Adibi et al. [21] studied the grinding force and machined surface quality of C_f_/SiC composites in dry grinding, MQL grinding and grinding with coolant. It showed that the minimum grinding force and wheel wear rate, as well as the maximum surface quality were obtained with MQL grinding process. The grinding ratio with MQL grinding was 115.38% higher than that with dry grinding. Qu et al. [22,23] investigated the effects of MQL on grinding of C_f_/SiC composites. A large amount of heat was removed by water vapor in the MQL grinding process, which reduced the grinding temperature significantly. Furthermore, effective oil films were formed in the contact areas between the grits and the material surface, which reduced the friction force and improved the grinding wheel life. Machining with cutting edge tools is regarded as a process that can achieve a higher material removal rate, better surface quality and size accuracy compared to grinding [24,25,26,27,28]. Diaz et al. [29,30] investigated the material removal mechanism in drilling of SiC_f_/SiC composites with a diamond-coated twist drill. It concluded that the fiber was fractured in a brittle way, and the matrix suffered a plastic-dominated mechanism. In addition, SiC particles were pulled out.

Hybrid machining is used to remove workpiece material with combined process mechanisms or energy sources. Among the hybrid machining methods, ultrasonic vibration-assisted machining has attracted wide attention because it has advantages of improved surface quality and machining efficiency, and reduced tool wear. Chen et al. [31] evaluated the machined surface integrity of C_f_/SiC composites processed with an ultrasonic-assisted milling method. The material was removed by micro brittle fracture, which was a result of the initiation and propagation of micro-cracks. The material removal was promoted by applying an appropriate tool amplitude. Bai et al. [32] proposed a novel coaxial helical gas-assisted laser water jet machining of SiC_f_/SiC composites. The effects of gas component and pressure on the stable length of water jet and surface water layer status were investigated. However, the material removal rate of the novel method was relatively low compared to the existing machining techniques such as laser irradiating and mechanical machining. Dong et al. [33] studied laser-assisted micromachining (LAMM) of SiC_f_/SiC composites, and tool wear and tool life in both LAMM and conventional micromachining were investigated, respectively. The results indicated that a maximum of 76% tool wear reduction and a maximum of 3.8-fold tool life improvement could be achieved with the LAMM process. Erdenechimeg et al. [34] deduced the optimal machining parameters in laser-assisted machining (LAM) of C_f_/SiC composites. The cutting force was decreased by about 40.7% with the LAM process compared to that with the conventional machining process under identical machining conditions.

According to the literature review, conventional machining methods, non-traditional machining methods, and hybrid machining methods have been applied to process fiber-reinforced SiC ceramic matrix composites. The grinding technique could achieve a fine surface finish, which is suitable for the finishing machining of the composites. However, the material removal rate of grinding is still low. Non-traditional machining techniques have advantages of no tool wear and high energy concentration. However, the machining efficiency is too low and the machined surface quality are deteriorated by the high energy. Hybrid machining techniques can improve the machining efficiency and surface quality. The milling method has the advantages of a high material removal rate and applicability to complex surface geometry. Up to now, no published literature on milling of SiC_f_/SiC composites and tool wear mechanisms can be found.

In this paper, PCD and CVD diamond tools have been used for high-speed milling of SiC_f_/SiC composite with a spindle speed of 10,000 rpm and feed speed of 3000 mm/min. Dry machining and cryogenic machining processes are carried out under identical milling parameters, respectively. Surface morphology, machining-induced defects and tool wear mechanisms are studied.

## 2. Experimental Procedure

### 2.1. Workpiece Material

**Figure 1:**
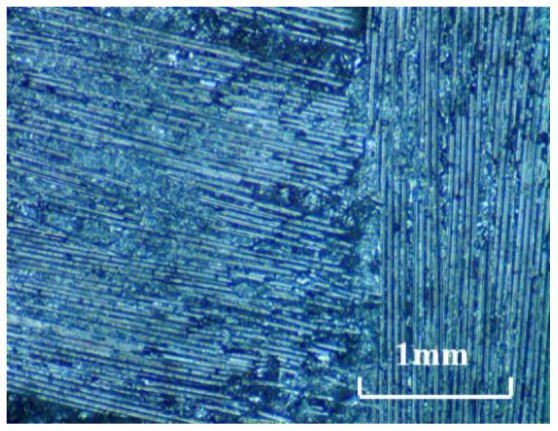
Surface morphology of the SiC_f_/SiC composite (×80).

The workpiece material is a SiC_f_/SiC composite (Fanrui Yihui Composite Material Co., Ltd., Zhengzhou, China) with 2.5 D braided structure, which is made up of SiC fibers and SiC matrix. The volume fraction of the fiber is about 30%. According to the different fiber directions, SiC fiber yarns are divided into warp yarns and weft yarns. The fiber yarns along the warp and weft direction are interwoven with each other in a form similar to the two-dimensional braided structure. The size of the workpiece blank is 200 × 20 × 5 mm^3^. Before the machining experiments, the workpiece blank is surface ground to ensure good flatness. The surface morphology of the SiC_f_/SiC composite is shown in Figure 1. The material properties supplied by the producer are listed in Table 1.

### 2.2. Cutting Tools

**Figure 2:**
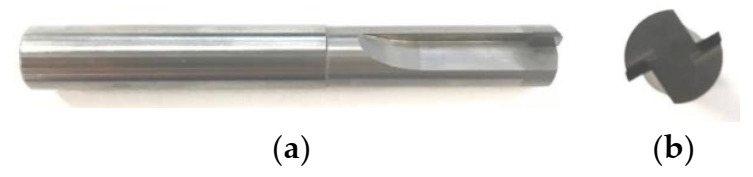
The CVD diamond milling cutter: (**a**) front view; (**b**) side view.

**Figure 3:**
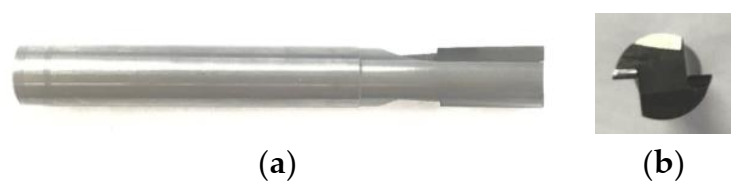
The PCD milling cutter: (**a**) front view; (**b**) side view.

In milling processing, the selection of tool material has a high impact on quality of the work material, tool life, and cost and efficiency of machining. In milling, the tool rotates with a high speed; thus, it is subjected to vibrations, high temperature, and friction. The tools used in this experimental investigation were two straight-toothed end-mill cutters. The cutting tools used cemented carbide as a shank. A polycrystalline diamond (PCD tool, RE1CR00CC-122R, Beijing Worldia Diamond Tools Co., Ltd., Beijing, China) and Chemical Vapor Deposition diamond (CVD diamond, RE1CR0000-122R, Beijing Worldia Diamond Tools Co., Ltd., Beijing, China) were used as blades. The thickness of the blades was 1 mm. The hardness of the PCD and CVD diamond blades is Hv7000 and Hv8000, respectively. The blades are brazed on the cemented carbide shank. The morphology of the CVD diamond and PCD tools are shown in Figure 2 and Figure 3, respectively. The diameter of the tool is 6 mm. The basic parameters of the milling tools provided by the supplier are shown in Table 2.

### 2.3. Experimental Setup

The machine tool used in the milling experiments was a DMU 60 mono BLOCK five-axis vertical machining center, from DMG, Germany. Through-slot milling experiments were performed on the SiC_f_/SiC composites. According to preliminary tests, the milling parameters used in this work are listed in Table 3. Firstly, milling experiments with both PCD and CVD diamond tools were performed under dry conditions. Secondly, cryogenic milling experiments with both PCD and CVD diamond tools were carried out. Tool wear and machined surface quality were investigated in both milling conditions.

**Figure 4:**
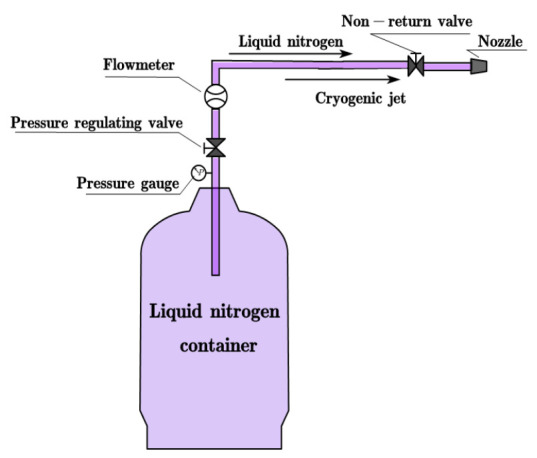
Cryogenic system.

The cryogenic cooling equipment (as shown in Figure 4) is composed of cryogenic liquid nitrogen container, pressure gauge, and valves. The liquid nitrogen with a pressure of 1.1 MPa was sprayed directly into the cutting area from the nozzle with a diameter of 4 mm. The tool wear morphology was observed by a camera-loaded microscope (UCMOS10000KPA CCD, Leica, Wetzlar, Germany). The surface morphology and micro defects of the milled workpiece were analyzed using a scanning electron microscope (S3400, Hitachi, Tokyo, Japan). In addition, the two-dimensional surface evaluation parameter *R*a cannot truly represent the surface quality because of the inherent pores inside the composites. Therefore, three-dimensional surface evaluation parameter *S*a is used to evaluate the machined surface quality. A 3D optical profiler (S Neox, Sensofar, Barcelona, Spain) was used to measure the *S*a of the machined surface.

## 3. Results and Discussions

### 3.1. Tool Wear

**Figure 5:**
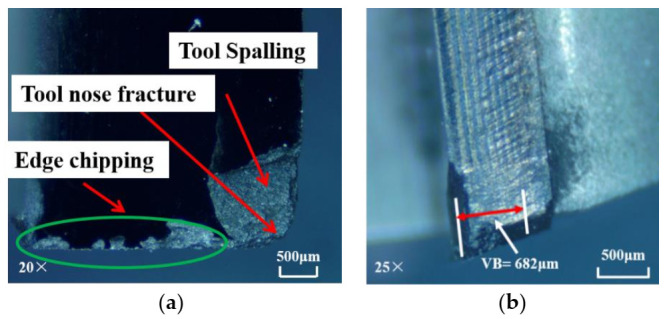
Wear of CVD diamond tool in dry machining: (**a**) rake face; (**b**) flank face (material removal amount = 21 mm^3^).

**Figure 6:**
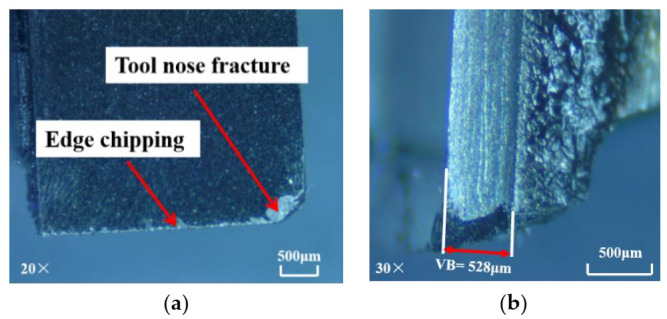
Wear of PCD tool in dry machining: (**a**) rake face; (**b**) flank face (material removal amount = 147 mm^3^).

Milling is an intermittent cutting process in which the cutting edge can easily collapse under the action of cyclic impact load. Both the PCD and CVD diamond tools used in this work are brittle materials, resulting in brittle fracture under cyclic impact load. When the critical load between the tool and the substrate is reached, the tool peels, leading to edge chipping or tool spalling. The wear of the CVD diamond tool, when used for machining under dry conditions, is portrayed in Figure 5. The wear magnitude is relatively high, and the rake surface of the diamond blade shows a large area of spalling, edge chipping, and severe tool nose fracture. However, when the PCD tool is applied in dry machining, its wear (as shown in Figure 6) is obviously smaller than that of the CVD diamond tool. Only a minute area of spalling around the tool nose is formed. The result implies that PCD cutters yield better performance than CVD diamond cutters when milling SiC_f_/SiC composites under dry conditions.

**Figure 7:**
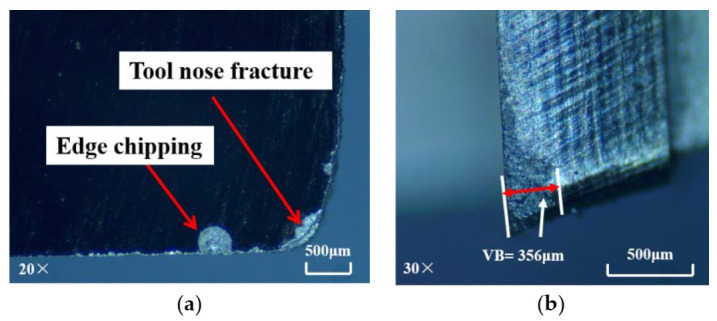
Wear of CVD diamond tool in cryogenic machining: (**a**) rake face; (**b**) flank face (material removal amount = 84 mm^3^).

**Figure 8:**
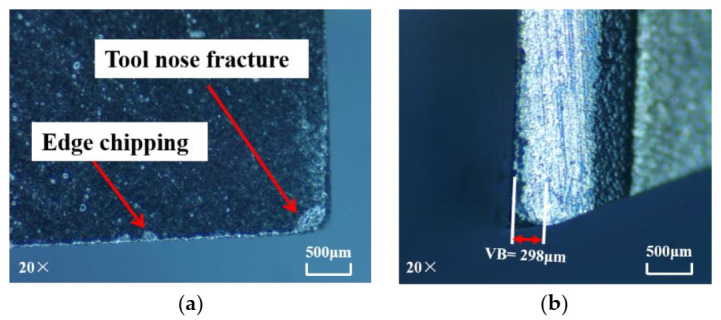
Wear of PCD tool in cryogenic machining: (**a**) rake face; (**b**) flank face (material removal amount = 294 mm^3^).

The SiC_f_/SiC composites are cooled by liquid nitrogen for 5 min before cryogenic machining experiments in order to ensure the accuracy of the experiments. In the preliminary experiments, the mechanical properties of the workpiece material under cryogenic conditions were tested and the results showed that the properties had no obvious change. Compared with dry machining, the wear of the CVD diamond tool has been greatly reduced, as shown in Figure 7. Low-magnitude wear is still apparent on the rake face of the cutter. Furthermore, minute spalling on the tool nose and edge chipping are also visible. Only a minute spalling is observable on the PCD tool, as shown in Figure 8. Thus, the wear condition of the PCD tool in cryogenic machining is better than that in dry machining, but the improvement of PCD tool wear under cryogenic conditions is not as obvious. Nevertheless, through analysis, it can be proved that the cryogenic machining plays a positive role in improving the wear condition of the tool.

The wear of the PCD tool and CVD diamond tool mainly occurred near the tool nose. This is because, during the cutting process, a portion of process heat dissipates while the other affects the surface of the work. The latter mainly influences the tool nose, raising its temperature above the other parts of the tool, rendering it more vulnerable to wear. In addition, the structure of SiC_f_/SiC composite materials is not uniform, and there are many fiber holes and hard spots in the material. Therefore, it is speculated that the large area of spalling on the rake face might have been caused by the high feed speed and impact force applied on the tool nose.

Although the hardness of the CVD diamond is higher than that of the PCD, its application is still limited by its weak fracture toughness [35,36], especially in interrupted machining such as milling. Due to the metallic binder of cobalt (Co) in PCD material, the PCD tool has higher fracture toughness than that of the CVD diamond tool. Milling is an interrupted process, resulting in a continuous strike on the cutting tool. As a result, CVD diamond tool with low fracture toughness and high brittleness would be broken easily, leading to tool failure in the form of edge chipping and tool spalling. On the other hand, the PCD tool has relatively higher fracture toughness, which makes it more suitable for the interrupted machining. This is also validated by [37]. Therefore, the tool wear of the PCD tool was slighter than that of the CVD diamond tool under both dry and cryogenic machining conditions.

### 3.2. Machined Surface Quality

**Figure 9:**
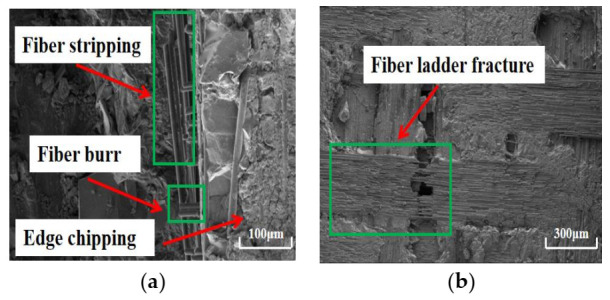
Morphology of the surface machined with CVD diamond tool in dry machining: (**a**) sidewall (×180); (**b**) bottom (×30).

**Figure 10:**
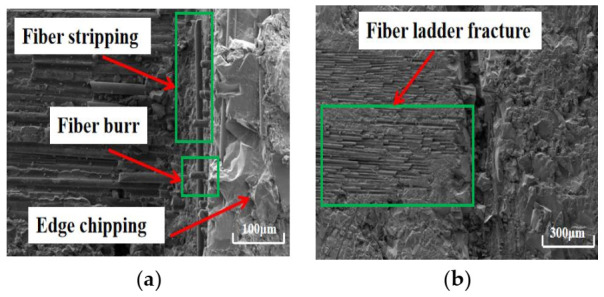
Morphology of the surface machined with PCD tool in dry machining: (**a**) sidewall (×220); (**b**) bottom (×70).

The surface quality of work material after dry machining and cryogenic machining has been compared. Under dry conditions, the bottom of the machined surface (as shown in Figure 9) processed by the CVD diamond tool appears to have undergone serious fiber ladder fracture. Moreover, a lot of fiber burring, fiber stripping, and edge chipping are also observed on the side wall of the machined surface processed by the PCD tool, as shown in Figure 10. The surface roughness *S*a of the bottom surfaces machined with CVD diamond tool and PCD tool are 5.4 μm and 4.9 μm, respectively. Furthermore, the bottom of the machined surface processed by the PCD diamond tool appears to have undergone serious fiber ladder fracture. In dry machining, there are many surface defects in the work, and the machining quality is poor.

**Figure 11:**
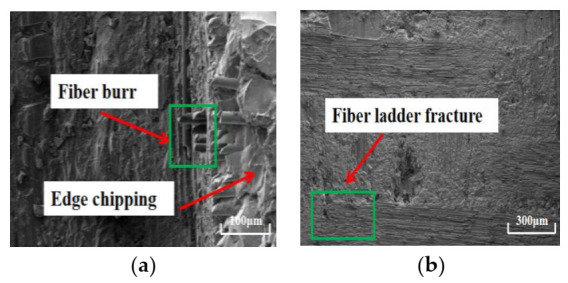
Morphology of the surface machined with CVD diamond tool in cryogenic machining: (**a**) sidewall (×300); (**b**) bottom (×30).

**Figure 12:**
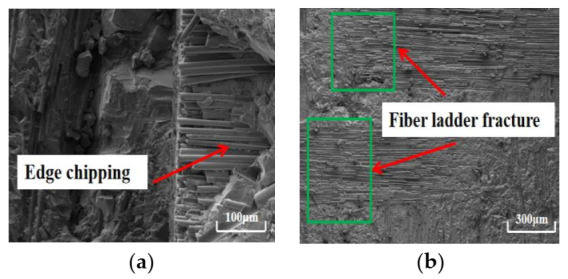
Morphology of the surface machined with PCD tool in cryogenic machining: (**a**) sidewall (×180); (**b**) bottom (×45).

Under cryogenic cooling, the surface quality of the bottom and sidewall of the machined surface processed by the CVD diamond tool and PCD tool is obviously better than that of the machined surface processed by dry machining. After processing with the CVD diamond tool, only a small amount of burr and edge chipping appears on the sidewall of the machined surface, and there is no fiber stripping in the sidewall of the machined surface—see Figure 11. After processing with the PCD tool, the sidewall of the machined surface (as shown in Figure 12) has no burr or fiber stripping, and the sidewall quality is greatly improved. However, after the CVD diamond tool and the PCD tool had performed machining, the fiber ladder fracture still existed on the bottom of the machined surface. However, in general, the integrity of the workpiece in cryogenic milling conditions is relatively better. The surface roughness *S*a of the machined surfaces processed with CVD diamond tool and PCD tool is 4.7 μm and 3.8 μm, respectively.

In the milling process, due to the high tensile strength and brittleness of SiC fiber, the axial fiber bundles are stretched under tensile stress. When the axial stress working on the fiber exceeds its tensile stress, fiber fracture takes place. Some of the broken fibers are extracted from the matrix, which causes ladder fracture at the bottom of the machined surface. The fibers on the side wall of the machined surface are cut off under the action of shear, extrusion, and bending fracture in the milling area. On the side wall surface of the machined surface, the fiber is subject to a greater axial force, which causes axial bending fracture rather than shear fracture. The bending fracture leads to the formation of burr. The strength of SiC matrix on the machined surface is insufficient, and the binding strength of SiC fiber is low, which leads to the peeling of SiC fiber and edge chipping on the side wall of the machined surface.

## 4. Conclusions

In this work, high-speed milling of SiC_f_/SiC composites under dry and cryogenic conditions with PCD and CVD diamond tools was investigated. Liquid nitrogen was utilized as the cooling medium in the cryogenic machining process. Straight slots with widths and depths of 6 mm and 3.5 mm, respectively, were machined under optimal processing parameters. The cutting performance of the two kinds of cutting tools was studied. The effects of cryogenic coolant on the tool wear and machined surface quality were investigated. The tool wear mechanisms and machined surface quality were analyzed. The conclusions obtained from this study were listed as follows:

(1) Under dry conditions, large pieces of spalling and edge chipping appeared on the rake face of the CVD diamond tool. On the other hand, minute edge chipping occurred on the PCD tool, and the tool wear was slighter than that of the CVD diamond tool. The PCD tool had better cutting performance than that of the CVD diamond tool. The wear on the rake face and cutting edge was a result of the continuous strike on the tool in the milling process.
(2) The application of cryogenic cooling resulted in a reduction of the wear on both the PCD and CVD diamond tools, leading to increased tool life values. The tool lives of the PCD and CVD diamond tools in cryogenic machining were two and four times higher than those in dry machining, respectively. The tool wear of the PCD end mill was still slighter than that of the CVD diamond end mill under identical milling and cryogenic cooling parameters. Owing to the metallic binder of cobalt (Co) in the PCD tool, the fracture toughness of the PCD tool was higher than that of the CVD diamond tool, resulting in slighter tool wear in machining SiC_f_/SiC composites.
(3) The material removal mechanisms mainly included fiber breakage, interface debonding and matrix brittle fracture. The main machining defects were fiber burr, fiber stripping, fiber ladder fracture, and edge chipping. The surface quality obtained under dry conditions was lower than that obtained under cryogenic conditions. This was a result of severe tool wear under dry conditions on both PCD and CVD diamond tools. Under cryogenic conditions, the surface quality machined with PCD tool was better than that machined with the CVD diamond tool.

Therefore, the PCD tool is more suitable for milling SiC_f_/SiC composites. In addition, in the milling of SiC_f_/SiC composites, cryogenic cooling can be used to reduce tool wear and improve work surface quality.

## Figures and Tables

**Table 1:** Properties of SiC_f_/SiC composite materials.

Parameter	Value
Density (g/cm^3^)	2.2–2.5
Tensile strength (MPa)	280–330
Elongation (%)	0.5–0.7
Young’s modulus (GPa)	190–210
Bending strength (MPa)	450–550

**Table 2:** Tool parameters.

Name	Blade Length (mm)	Rake Angle (°)	Clearance Angle (°)	Total Length (mm)
PCD tool	20	3	10	100
CVD diamond tool	4	0	5	80

**Table 3:** Experimental parameters.

Milling Parameters	Parameter Value
Spindle speed *n* (rpm)	10,000
Feed speed *v*_f_ (mm/min)	3000
Cutting width *a*_e_ (mm)	6
Cutting depth *a*_p_ (mm)	0.5

## Data Availability

Not applicable.

## References

[1] Igor P., Nikolay R., Dmitriy M., Alexey S., Yuliya L., Anatoly P. (2020). Fabrication of Silicon Carbide Fiber-Reinforced Silicon Carbide Matrix Composites Using Binder Jetting Additive Manufacturing from Irregularly-Shaped and Spherical Powders. Materials.

[2] Choi J.H., Nam Y.W., Jang M.S., Kim C.G. (2018). Characteristics of silicon carbide fiber-reinforced composite for microwave absorbing structures. Compos. Struct..

[3] Yang L.W., Liu H.T., Cheng H.F. (2017). Processing-temperature dependent micro- and macro-mechanical properties of SiC fiber reinforced SiC matrix composites. Compos. Part B.

[4] Chai Y.X., Zhou X.G., Zhang H.Y. (2017). Effect of oxidation treatment on KD–II SiC fiber–reinforced SiC composites. Ceram. Int..

[5] Cheng T.B., Wang X.R., Zhang R.H., Pei Y.M., Ai S.G., He R.J., Fang D.N., Yang Y.Z. (2020). Tensile properties of two-dimensional carbon fiber reinforced silicon carbide composites at temperatures up to 2300 °C. J. Eur. Ceram. Soc..

[6] Albert J.S., Berend D., Thilo G., David C., Hong H.C., Tsai H.Y., Hitoshi O., Kazutoshi K., Pei Z.J. (2018). Fixed abrasive machining of non-metallic materials. CIRP Ann. Manuf. Technol..

[7] An Q.L., Chen J., Ming W.W., Chen M. (2021). Machining of SiC ceramic matrix composites: A review. Chin. J. Aeronaut..

[8] Xu L., Zhao G.L., Zhang J.Q., Wang K., Wang X.Y., Hao X.Q. (2020). Feasibility study on cryogenic milling of carbon fiber reinforced silicon carbide composites. Trans. Nanjing Univ. Aeronaut. Astronaut..

[9] Liu Y.S., Zhang R.H., Li W.N., Wang J., Yang X.J., Cheng L.F., Zhang L.T. (2018). Effect of machining parameter on femtosecond laser drilling processing on SiC/SiC composites. Int. J. Adv. Manuf. Technol..

[10] Li W.N., Zhang R.H., Liu Y.S., Wang C.H., Wang J., Yang X.J., Cheng L.F. (2016). Effect of different parameters on machining of SiC/SiC composites via pico-second laser. Appl. Surf. Sci..

[11] Zhai Z.Y., Wei C., Zhang Y.C., Cui Y.H., Zeng Q.R. (2020). Investigations on the oxidation phenomenon of SiC/SiC fabricated by high repetition frequency femtosecond laser. Appl. Surf. Sci..

[12] Zhao J., Wang W.J., Wang R.J., Cui J.L. (2018). Machining millimeter-scale deep holes in SiC_f_/SiC material using femtosecond laser filamentation effect. Mater. Sci. Adv. Compos. Mater..

[13] Liu C., Zhang X.Z., Gao L., Jiang X.G., Li C., Yang T. (2021). Feasibility of micro-hole machining in fber laser trepan drilling of 2.5D C_f_/SiC composite: Experimental investigation and optimization. Opt. Int. J. Light Electron. Opt..

[14] Chen J., An Q.L., Ming W.W., Chen M. (2021). Investigations on continuous-wave laser and pulsed laser induced controllable ablation of SiC_f_/SiC composites. J. Eur. Ceram. Soc..

[15] Chou K.J., Usami H., Enomoto K. (2018). Micro abrasive jet machining of silicon carbide (SiC) fiber reinforced ceramic matrix composite. Adv. Mater. Res..

[16] Wei C.J., Zhao L., Hu D.J., Ni J. (2013). Electrical discharge machining of ceramic matrix composites with ceramic fiber reinforcements. Int. J. Adv. Manuf. Technol..

[17] Yue X.M., Li Q., Yang X.D. (2020). Influence of thermal stress on material removal of C_f__SiC composite in EDM. Ceram. Int..

[18] Yin J.F., Xu J.H., Ding W.F., Su H.H. (2021). Effects of grinding speed on the material removal mechanism in single grain grinding of SiC_f_/SiC ceramic matrix composite. Ceram. Int..

[19] Cao X.Y., Lin B., Zhang X.F. (2013). A study on grinding surface waviness of woven ceramic matrix composites. Appl. Surf. Sci..

[20] Zhang L.F., Wang S., Li Z., Qiao W.L., Wang Y., Wang T. (2019). Influence Factors on Grinding Force in Surface Grinding of Unidirectional C/SiC Composites. Appl. Compos. Mater..

[21] Adibi H., Esmaeili H., Rezaei S.M. (2018). Study on minimum quantity lubrication (MQL) in grinding of carbon fiber-reinforced SiC matrix composites (CMCs). Int. J. Adv. Manuf. Technol..

[22] Qu S.S., Gong Y.D., Yang Y.Y., Sun Y., Wen X.L., Qi Y. (2020). Investigating Minimum Quantity Lubrication in Unidirectional C_f_/SiC composite grinding. Ceram. Int..

[23] Qu S.S., Gong Y.D., Yang Y., Wang W., Liang C.Y., Han B. (2020). An investigation of carbon nanofluid minimum quantity lubrication for grinding unidirectional carbon fibre-reinforced ceramic matrix composites. J. Clean. Prod..

[24] Zhang X.W., Yu T.B., Li M., Wang Z.X. (2020). Effect of machining parameters on the milling process of 2.5D C/SiC ceramic matrix composites. Mach. Sci. Technol..

[25] Hu M., Ming W.W., An Q.L., Chen M. (2019). Experimental study on milling performance of 2D C/SiC composites using polycrystalline diamond tools. Ceram. Int..

[26] Bao Y.J., Bi M.Z., Gao H., Cao B. (2013). Effect of Fiber Directions on the Surface Quality of Milling C/SiC Composites. Adv. Mater. Res..

[27] Ding K., Fu Y.C., Su H.H., Chen Y., Yu X.Z., Ding G.Z. (2014). Experimental studies on drilling tool load and machining quality of C/SiC composites in rotary ultrasonic machining. J. Mater. Process. Technol..

[28] Xing Y.Q., Deng J.X., Zhang G.D., Wu Z., Wu F.F. (2017). Assessment in drilling of C/C-SiC composites using brazed diamond drills. J. Manuf. Process..

[29] Gavalda Diaz O., Axinte D.A., Novovic D. (2018). Probabilistic modelling of tool unbalance during cutting of hard- heterogeneous materials: A case study in Ceramic Matrix Composites (CMCs). Compos. Part B.

[30] Gavalda Diaz O., Axinte D.A., Butler-Smith P., Novovic D. (2019). On understanding the microstructure of SiC/SiC Ceramic Matrix Composites (CMCs) after a material removal process. Mater. Sci. Eng. A.

[31] Chen J., Ming W.W., An Q.L., Chen M. (2020). Mechanism and feasibility of ultrasonic-assisted milling to improve the machined surface quality of 2D C_f_/SiC composites. Ceram. Int..

[32] Cheng B., Ding Y., Li Y., Li J.Y., Xu J.J., Li Q., Yang L.J. (2021). Coaxial helical gas assisted laser water jet machining of SiC/SiC ceramic matrix composites. J. Mater. Process. Technol..

[33] Dong X.Y., Shin Y.C. (2017). Improved machinability of SiC/SiC ceramic matrix composite via laser-assisted micromachining. Int. J. Adv. Manuf. Technol..

[34] Erdenechimeg K., Jeong H.I., Lee C.M. (2019). A Study on the Laser-Assisted Machining of Carbon Fiber Reinforced Silicon Carbide. Materials.

[35] Inspektor A., Oles E.J., Bauer C.E. (1997). Theory and Practice in Diamond Coated Metal Cutting Tools. Int. J. Refract. Met. Hard Mater..

[36] Sun F.H., Zhang Z.M., Chen M., Shen H.S. (2003). Improvement of adhesive strength and surface roughness of diamond films on Co-cemented tungsten carbide tools. Diam. Relat. Mater..

[37] Yan G., Yue W., Meng D.Z., Lin F., Wu Z.Y., Wang C.B. (2016). Wear performances and mechanisms of ultrahard polycrystalline diamond composite material grinded against granite. Int. J. Refract. Met. Hard Mater..

